# Effects of Aging and Disease Conditions in Brain of Tumor-Bearing Mice: Evaluation of Purine DNA Damages and Fatty Acid Pool Changes

**DOI:** 10.3390/biom12081075

**Published:** 2022-08-04

**Authors:** Marios G. Krokidis, Paraskevi Prasinou, Eleni K. Efthimiadou, Andrea Boari, Carla Ferreri, Chryssostomos Chatgilialoglu

**Affiliations:** 1 Istituto per la Sintesi Organica e la Fotoreattività, Consiglio Nazionale delle Ricerche, Via Piero Gobetti 101, 40129 Bologna, Italy; 2 Institute of Nanoscience and Nanotechnology, National Center for Scientific Research “Demokritos”, 15310 Athens, Greece; 3 Faculty of Veterinary Medicine, University of Teramo, 64100 Teramo, Italy; 4 Department of Chemistry, National and Kapodistrian University of Athens, 15784 Athens, Greece; 5 Center for Advanced Technologies, Adam Mickiewicz University, 61-614 Poznan, Poland

**Keywords:** tumor-bearing mice, aging, hydroxyl radical, oxidatively-induced DNA lesions, brain fatty acids, age-induced remodeling

## Abstract

The consequences of aging and disease conditions in tissues involve reactive oxygen species (ROS) and related molecular alterations of different cellular compartments. We compared a murine model of immunodeficient (SCID) xenografted young (4 weeks old) and old (17 weeks old) mice with corresponding controls without tumor implantation and carried out a compositional evaluation of brain tissue for changes in parallel DNA and lipids compartments. DNA damage was measured by four purine 5′,8-cyclo-2′-deoxynucleosides, 8-oxo-7,8-dihydro-2′-deoxyguanosine (8-oxo-dG), and 8-oxo-7,8-dihydro-2′-deoxyadenosine (8-oxo-dA). In brain lipids, the twelve most representative fatty acid levels, which were mostly obtained from the transformation of glycerophospholipids, were followed up during the aging and disease progressions. The progressive DNA damage due to age and tumoral conditions was confirmed by raised levels of 5′*S*-cdG and 5′*S*-cdA. In the brain, the remodeling involved a diminution of palmitic acid accompanied by an increase in arachidonic acid, along both age and tumor progressions, causing increases in the unsaturation index, the peroxidation index, and total TFA as indicators of increased oxidative and free radical reactivity. Our results contribute to the ongoing debate on the central role of DNA and genome instability in the aging process, and on the need for a holistic vision, which implies choosing the best biomarkers for such monitoring. Furthermore, our data highlight brain tissue for its lipid remodeling response and inflammatory signaling, which seem to prevail over the effects of DNA damage.

## 1. Introduction

**Figure 1:**
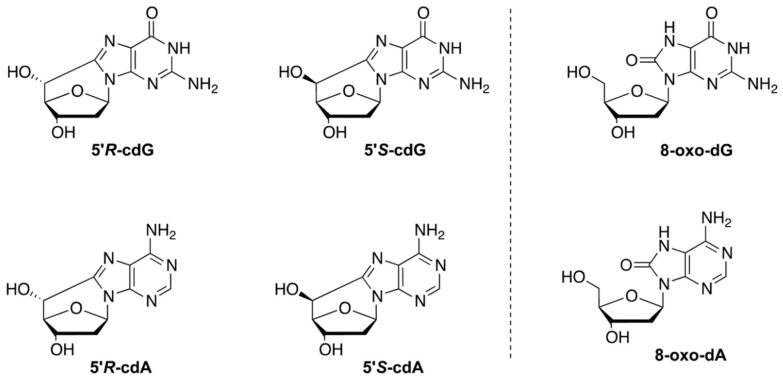
Structures of 5′,8-cyclo-2′-deoxyguanosine (cdG) and 5′,8-cyclo-2′-deoxyadenosine (cdA) in their 5′*R* and 5′*S* diastereomeric forms (**left**) and 8-oxo-7,8-dihydro-2′-deoxyguanosine (8-oxo-dG) and 8-oxo-7,8-dihydro-2′-deoxyadenosine (8-oxo-dA) (**right**).

The aging process or disease conditions cause free radical stress and can impair the molecular and enzymatic network that controls the redox balance in organisms [1,2]. Single DNA adducts or multiple lesions generated under stress, such as tandem or clustered lesions, are substrates of the different cellular repair systems that protect genome instability. However, due to the progressive loss of the protective machineries, depending either on the aging degenerative mechanisms or on enzymatic deficiencies, these adducts are poorly repaired and, therefore, may accumulate in the genome, causing damage to cellular components [3]. Purine 5′,8-cyclo-2′-deoxynucleosides (cPu) are solely generated by the attack of HO^•^ radicals on purine moiety via C5′-radical chemistry, resulting in the formation of an additional C5′−C8 covalent bond; 5′,8-cyclo-2′-deoxyadenosine (cdA) and 5′,8-cyclo-2′-deoxyguanosine (cdG) exist in the 5′*R* and 5′*S* configurations (Figure 1) [4,5,6]. cPu can be removed only by the nucleotide excision repair (NER) pathway, and different repair efficiencies of the *R* and *S* diastereoisomers has been detected [7,8]. On the contrary, the well-known 8-oxo-purines (8-oxo-Pu) lesions, i.e., 8-oxo-7,8-dihydro-2′-deoxyadenosine (8-oxo-dA) and 8-oxo-7,8-dihydro-2′-deoxyguanosine (8-oxo-dG), are derived from the oxidation at the C8 position of adenine and guanine by a variety of reactive oxygen species (ROS) and can be repaired by base excision repair (BER) [9]. The role of oxygen is crucial in the formation of the four cPu. In recent model studies by our group [10,11], cPu levels decreased substantially by increasing oxygen tension, favoring products derived from the peroxyl radical. It should be emphasized that 8-oxo-Pu gradually increased by increasing oxygen concentration. Interestingly, a similar trend was not reported in a cellular environment, where reducing the oxygen incubation conditions raises the accumulation of both cPu and 8-oxo-Pu [12,13]. The brain presents an increased oxidative stress due to a high level of tissue oxygen consumption and a diminished ratio of antioxidant to pro-oxidant enzymes [14]. An age-dependent accumulation, with much higher levels of cdA, was observed in the brain as well as in liver and kidney of (XPA)-deficient mice, compared with wild-type animals, showing the strong involvement of NER in the effective repair of oxidative DNA damage in different tissue compartments [15].

**Figure 2:**
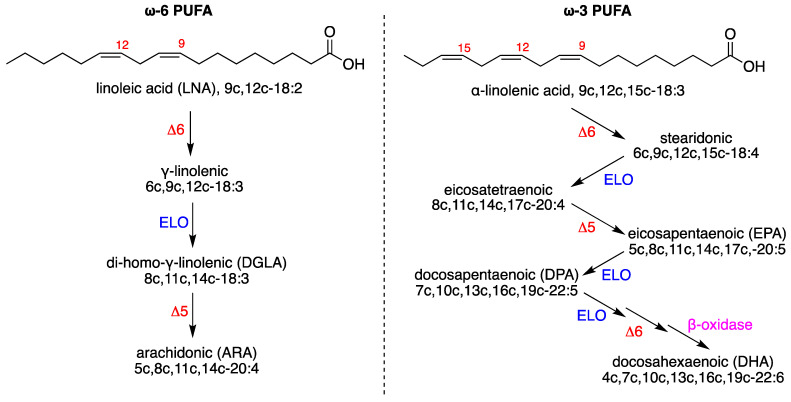
Biosynthesis of PUFA: (**left**) the omega-6 series starting linoleic acid; (**right**) the omega-3 series starting alpha-linolenic acid. Enzymes: ELO elongase; Δ5-, Δ6-, and Δ9-desaturase; β-oxidase. Numerical abbreviations describing the position and cis geometry of the double bonds (e.g., 9c), as well as the notation of the carbon chain length and total number of unsaturations (e.g., 18:2); in parentheses are the acronyms used in this work (e.g., ARA for arachidonic acid).

Another important molecular contribution in the brain, under physiological and pathological conditions, comes from lipid homeostasis, which is strongly connected with chemical events due to the redox properties of several reactive species that can cause oxidative damage [16]. The brain is the tissue that is second-most rich in lipids, after adipose tissue, with necessary components that support structural, biochemical, and cell signaling functions. Its homeostasis corresponds to the correct exploitation of the various processes, including immunity, inflammation, and resolution. The phospholipid composition of neuronal and glial membranes in mammals, with about 75% of lipids exclusive to neural tissue, is a clear example of the role of the molecular characteristics that are needed to accomplish the wide variety of organization and shaping of brain tissue [17]. Changes in brain lipidome are reported in the modulation of neuronal functions, as well as in neurodegenerative diseases and aging, with glycerophospholipids, sphingolipids, and sterols representing the majority of the brain’s lipids [18,19]. A fatty acid-based analysis of neural tissue showed evidence of the presence of saturated and monounsaturated fatty acids (SFA and MUFA) that are formed by de novo lipogenesis, together with polyunsaturated fatty acids (PUFA), which are supplied to neuronal cells by an exchange with blood lipids and biosynthesized in the brain cells after the intakes of essential PUFAs, i.e., linoleic acid (18:2 omega-6, LNA) and alpha-linolenic acid (18:3, omega-3), which cannot be formed in mammals (Figure 2) [20]. However, in rodents, the PUFA biosynthesis is not very active in the brain [21], which should receive a constant supply. Especially important for the correct brain functioning is the balance between the omega-6 PUFA arachidonic acid (ARA) and the omega-3 PUFA docosahexaenoic acid (DHA) in neuronal membrane phospholipids, to ensure the appropriate release of these fatty acids as precursors of lipid mediators with pro- and anti-inflammatory properties [20,21,22]. The occurrence of inadequate levels due to diet or consumption, resulting from oxidative damage to PUFA, is the basis for important neuronal impairments, as described in seminal papers on neuronal membrane expansion [23], cognitive development [24], Alzheimer’s disease, and Parkinson disease [25,26,27,28].

In this scenario of molecular contributions to tissue health, oxidation cannot be seen as having a damaging effect, because oxidative metabolism in the brain is necessary, with 20% of the total basal oxygen budget used to support not only the ATP cellular production but also the production of reactive oxygen species and oxygenated metabolites, allowing for adaptation and signaling in neuronal tissues, as described in a recent review [29]. Therefore, molecular damage must be carefully evaluated in order to distinguish between damage and signals. The role of thiols is crucial, either as sulfur compounds—including the amino acid cysteine—or as a functional group in enzymes, as the thiol–disulfide redox balance allows the quenching of ROS reactivity, as shown for glutathione in Alzheimer’s patients and in a Parkinson animal model [30,31].

In our holistic vision of oxidative stress, we explored specific biomarkers of free radical damage. In particular, for biomarkers for DNA reactivity, we envisaged a specific radical-based reaction of purine nucleosides that are transformed into cPu and 8-oxo-Pu lesions [4,5,6], and for biomarkers of membrane reactivity, we envisaged both the formation of trans fatty acids (TFA) and the remodeling of membrane lipidome as interesting markers of tissue transformation [32,33,34]. In TFA formation, thiyl radicals generated from the antioxidant reactivity of thiols are involved in the addition-elimination reaction on the unsaturated double bonds of MUFA and PUFA. In membrane remodeling, MUFA and PUFA residues respond to different conditions, such as aging or disease progression, and their balance can be helpful in distinguishing between free radical or metabolic events in the specific pathological condition in models and clinical studies [35,36].

We recently used an experimental diseased animal model—severe combined immunodeficient (SCID) female mice inoculated with U87MG human glioblastoma cells—to assess differences in the cPu levels of the liver and the kidney at the early and final stages of tumorigenesis (4-week-old and 17-week-old tumor-bearing mice, respectively). A profound enrichment of oxidatively induced DNA damage lesions in both tissues of 17-week-old xenografts, compared with the early stage of tumorigenesis, was observed [37]. In the present paper, we report the examination of brain tissue coupling for the content of both cPu and 8-oxo-Pu with those of fatty acid components in the brains of young and older tumor-bearing mice, with a parallel evaluation of the same lesions in control SCID mice without tumor implantation (4 weeks old and 17 weeks old, respectively). The aim of our work is to unravel potential differences in tumor-bearing animals and to obtain important insights that are related to immunodeficiency and aging.

## 2. Materials and Methods

### 2.1. Materials

All reagents were obtained from Sigma–Aldrich (Steinheim, Germany) and solvents (chloroform, methanol, n-hexane) were purchased from Fisher Scientific (HPLC grade). Nuclease P1 from Penicillium citrinum, phosphodieasterase I and II, alkaline phosphatase from bovine intestinal mucosa, DNase I and DNase II, benzonase 99%, BHT, deferoxamine mesylate, and pentostatin were purchased from Sigma-Aldrich (Steinheim, Germany). RNase T1 was obtained from Thermo Fisher Scientific (Waltham, MA, USA) and RNase A was obtained from Roche Diagnostic GmbH (Mannheim, Germany). 2′-Deoxyadenosine monohydrate were purchased from Berry & Associates Inc. (Dexter, NY, USA). All fatty acid methyl esters (FAME) used as references were commercially available from Supelco (Bellefonte, PA, USA) or Sigma–Aldrich. U87MG brain glioblastoma was obtained from the American Type Culture Collection (ATCC). High glucose Dulbecco’s modified Eagle Medium (DMEM) was purchased from Sigma. Trypsin-EDTA, L-glutamine, penicillin–streptomycin solution, and heat inactivated fetal bovine serum (FBS) were obtained from Biochrom KG. Ultrapure water (18.3 MΩ·cm) and deionized water (Milli-Q water) were purified by a Milli-Q system (Merck–Millipore, Bedford, PA, USA).

### 2.2. Animal Studies and Xenografts Construction

Female SCID and normal Swiss mice were housed at the SOL-GEL laboratory at the NCSR “Demokritos” and SCID mice were xenografted at two weeks of age, subcutaneously just above the right flank, with U87MG cells that were previously grown in DMEM, as previously described [37]. The tumor volume, mice weights, and survival rates were calculated in different time intervals. Mice were housed in groups of three per cage under positive pressure in polysulfone type IIL individual ventilated cages (Sealsafe, Tecniplast, Buguggiate, Italy) and had ad libitum access to water and food. Room temperature and relative humidity were 24 ± 2 °C and 55 ± 10% respectively. All animals in the facility were screened regularly according to the Federation of European Laboratory Animal Science Associations’ recommendations and were found to be free of the respective pathogens. Animals were sacrificed under deep ether anesthesia and the brain tissues were rapidly extracted, placed in a polypropylene tube, immediately snap-frozen in liquid nitrogen, and stored at −80 °C.

### 2.3. DNA Isolation and Quantification of Modified Nucleosides by Stable Isotope Dilution LC-MS/MS

Genomic DNA from frozen tissues was isolated using a high-salt extraction procedure [37,38], enzymatically digested in the presence of 100 μΜ deferoxamine, 100 μΜ butylated hydroxytoluene. And the internal standards ([^15^N_5_]-5′*S*-cdA, [^15^N_5_]-5′*R*-cdA, [^15^N_5_]-5′*S*-cdG, [^15^N_5_]-5′*R*-cdG, [^15^N_5_]-8-oxo-dG and [^15^N_5_]-8-oxo-dA), and lesions were quantified as described previously [4,5,6,10,11,12,13,39]. The samples were filtered by centrifugation through a 3 kDa microspin filter (Millipore; Bedford, OH, USA), cleaned and enriched by an HPLC-UV system coupled with a sample collector, and injected into the LC-MS/MS system. The quantification of the modified nucleosides was carried out by a triple-stage quadrupole mass spectrometer using positive electrospray ionization (ESI), following a gradient program (2 mM ammonium formate, acetonitrile, and methanol), and the detection was executed in multiple reaction monitoring mode (MRM) using the two most intense and characteristic precursor/product ion transitions for each lesion [11,38].

### 2.4. Fatty Acid Analysis of Brain Tissues

Tissues were disrupted in 2:1 chloroform:methanol using a mechanical homogenizer submerged in a cooling bath of acetone and dry ice to maintain a temperature of approximately −80 °C. The lipid extract, after solvent evaporation to dryness, was then treated with 0.5 M KOH/MeOH for 10 min at room temperature under stirring for the derivatization of fatty acid residues of the glycerol esters-containing lipids into their corresponding fatty acid methyl esters (FAME) [40]. After this transesterification step, FAME were extracted with n-hexane; n-hexane phase was dehydrated with anhydrous Na_2_SO_4_, evaporated and analyzed via an Agilent 7890B CG system equipped with a 60 m × 0.25 mm × 0.25 μm (50%-cyanopropyl)-methylpolysiloxane column (DB23, Agilent, USA) and a flame ionization detector (FID), with an injector temperature at 230 °C and split injection of 50:1. Oven temperature started at 165 °C, was held for 3 min, then increased by 1 °C/min up to 195 °C, held again for 40 min, then increased by 10 °C/min up to 240 °C and held for 10 min. A constant pressure mode (29 psi), with helium as the carrier gas, was used. Methyl esters were identified by comparison with the retention times of commercially available standards or trans fatty acid references, which were obtained as described elsewhere [41,42].

### 2.5. Statistical Analysis

The mice groups were divided into groups of six and the results were expressed as mean ± standard deviation (SD). The statistical significance (*p* values) of the results was calculated by unpaired two-tailed Student’s t-test using GraphPad Prism™ software version 6.01 for Windows (GraphPad Software Inc., La Jolla, CA, USA). A multiple comparison test was applied to compare the differences among the distinct pairs of groups.

## 3. Results

### 3.1. Protocol Outline

Human tumor xenografts were obtained by inoculating U87MG human brain glioblastoma cells subcutaneously in two-week-old SCID mice. Approximately 2 weeks post-injection, the first set of animals was sacrificed (at 4 weeks of age). The second set of tumor-bearing mice was sacrificed at 17 weeks of age. In this article, the first group is referred as being in the early stage of tumorigenesis, while the second group is referred to as being in the final stage of tumor presence and characterized by very poor condition.

We also evaluated DNA lesions and the fatty acids pool in the brain tissue of the control SCID mice without tumor implantation, at 4 weeks of age and 17 weeks of age, to identify their potential differences with tumor-bearing animals. Because human xenograft were selected and the tumors originated by way of exogenous inoculation with human malignant cells, the tumor tissues were not analyzed, following an approach described for a genetically engineered mouse [43]. The accumulation of DNA damage in tissues that are distant from a tumor site can be induced by tumors of different origin, sometimes as a consequence of a cancer-related chronic inflammatory response in vivo [44]. Parallel DNA and lipid analyses were also performed in normal Swiss mice to evaluate healthy conditions as well as to recognize the potent differences between a healthy state and an immune-deficient condition.

Genomic DNA was isolated from the brains, hydrolyzed to single nucleosides by an enzymatic cocktail containing nucleases, and analyzed by LC-MS/MS for the determination of the modified nucleosides (the four cPu and two 8-oxo-Pu). The evaluation of the fatty acid pool was performed in the lipid extract, under conditions in which the fatty acid esterified to glycerol moieties (mainly brain glycerophospholipids) were transformed to fatty acid methyl esters (FAMEs) and could be analyzed by GC, as described elsewhere (for details, see the Materials and Methods section).

### 3.2. Evaluation of Purine DNA Lesions

**Figure 3:**
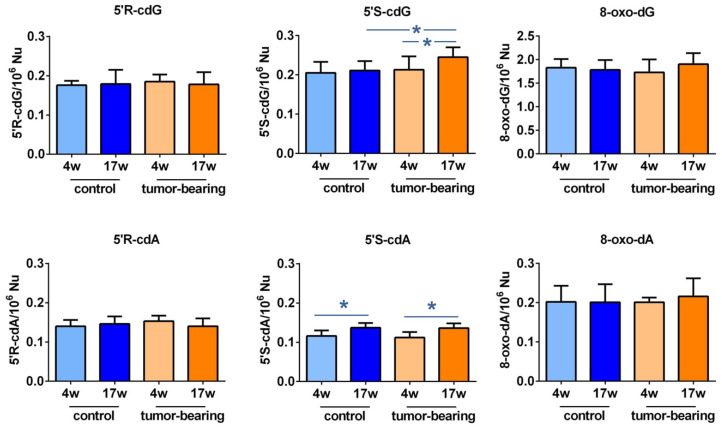
Purine DNA lesions in the brain of SCID mice: the levels (lesions/10^6^ nucleosides) of 5′*R*-cdG, 5′*S*-cdG, 5′*R*-cdA, 5′*S*-cdA, 8-oxo-dG, and 8-oxo-dA in the brain tissue of control SCID and tumor-bearing SCID mice. For specific values, see Tables S1 and S2. The values are given as mean ± SD (*n* = 6); * (*p* < 0.05).

An LC-MS/MS analysis of the brain DNA nucleosides provided the four cPu and the two 8-oxo-Pu lesions of our animal cohorts. In general, as shown in Table 1 and Figure 3, the levels of cPu and 8-oxo-dA were found to be similar, while 8-oxo-dG was one order of magnitude higher (for specific values for normal mice, see Table S1). Examining the SCID groups, we found the following: (i) a significant enhancement of 5′*S*-cdG in 17-week-old xenografted mice, compared with control SCID animals of the same age (*p* = 0.0370, cf. Table S2 for *p*-values); (ii) increased levels of 5′*S*-cdG in 17-week-old control SCID mice, compared with younger xenografts ((*p* = 0.0354); (iii) 5′*S*-cdA was significantly raised in old tumor-bearing SCID mice, compared with the 4-week-old ones (*p* = 0.0342); and (iv) 5′*S*-cdA was also at higher levels in 17-week-old control animals, compared to 4-week-old ones (*p* = 0.0102).

**Figure 4:**
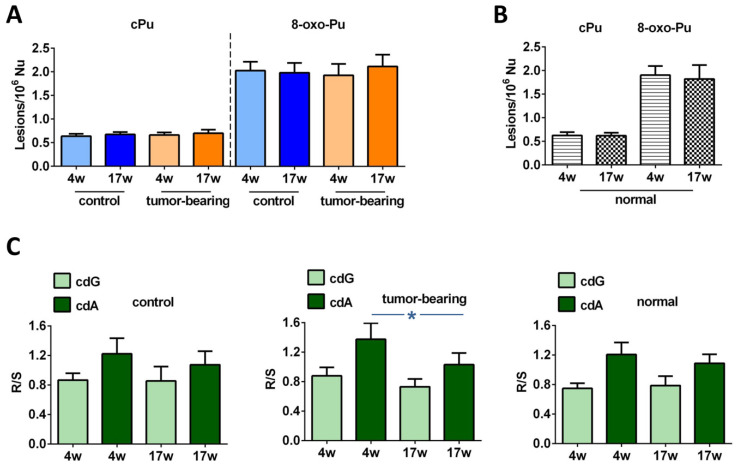
LC-MS/MS analysis of purine DNA lesions. (**A**) The levels (lesions/10^6^ nucleosides) of cPu and 8-oxo-Pu in the brain tissue of control SCID and tumor-bearing SCID mice; for specific values see Tables S3 and S4. (**B**) The levels (lesions/10^6^ nucleosides) of cPu and 8-oxo-Pu in the brain tissue of normal Swiss mice; for specific values see Tables S3 and S4. (**C**) The 5′*R*/5′*S* ratio of cdG and cdA in control SCID, tumor-bearing SCID, and normal Swiss mice; for specific values see Tables S5 and S6. The values are given as mean ± SD (*n* = 6); * (*p* < 0.05).

Examining the effects of age and tumor progression in our SCID cohorts, alterations that were not statistically significant were exhibited at the levels of total cPu and 8-oxo-Pu, as highlighted in Figure 4A (for specific values, see Tables S3 and S4). To estimate the healthy conditions, Swiss mice were examined for their brains’ accumulation of DNA lesions at the ages of 4 weeks and 17 weeks, to show whether any significant change was present (Figure 4B and Table S4; see also Figure S1 and Tables S1 and S2 for the single lesions). Notably, a comparison between the Swiss mice and the control SCID mice showed evidence of a significant enhancement of 5′*S*-cdA at 17 weeks of age in immunodeficient animals (*p* = 0.0049, Table S2). In general, the Swiss mice presented with similar levels of cPu and with lower 8-oxo-Pu levels, although the difference was not significant, compared with control SCID mice (Tables S3 and S4).

Regarding the ratio of diastereoisomeric lesions of cPu, it was reported that this indicator provided important information on the structural conformation of both isomers associated with the repair process [7,8]. The 5′*R*/5′*S* ratio of cdA was found to be higher than the ratio of corresponding cdG in all groups of samples, as shown in Figure 4C. A decrease in the *R*/*S* ratio of cdG and a significant diminution of the *R*/*S* ratio of cdA (*p* = 0.0211) were noted in 17-week-old tumor-bearing mice, compared with younger xenograted animals (Tables S5 and S6). Differences that were not statistically significant were observed in the control SCID as well as in the normal Swiss animals (Tables S5 and S6).

### 3.3. Evaluation of Brain Fatty Acid Pool

**Figure 5:**
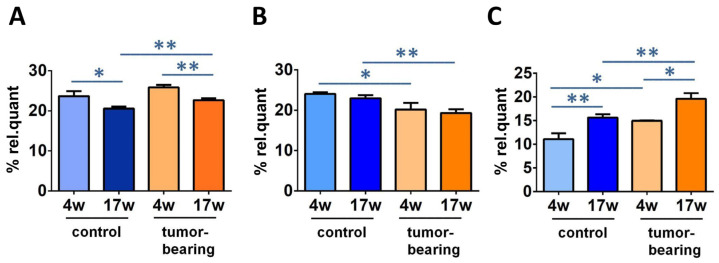
Some relevant fatty acid changes in SCID mice cohorts. Comparison of % rel. quant. of (**A**) C16:0; (**B**) 9c-18:1; (**C**) ARA in the brain tissue among control SCID mice and tumor-bearing SCID mice at 4 weeks of age and 17 weeks of age (for specific values, see Table 2). Significance: * (*p* < 0.05), ** (*p* < 0.01).

After isolation of the brain lipid content, we proceeded to the transformation of the fatty acid-containing lipids to their fatty acid methyl ester (FAME) derivatives, as described in the Materials and Methods section of this article, following a previously reported procedure [41,42]. The FAME separation and identification was carried out by the gold standard of gas chromatography (GC) analysis under known conditions, and we focused our attention to 12 fatty acid methyl esters (corresponding to >97% of the peaks that were present in the analysis), which were calibrated using commercially available materials for quantitative purposes. The values of this fatty acid cluster are reported in Table 2 as relative quantitative percentages (% rel. quant.), obtained by the quantitative data and reported as percentages of each FAME over the total FAME quantities resulting from the GC areas, following a known procedure (see Table S7 for the *p*-values of Table 2) [42]. The relevant results in our cohorts are reported in Figure 5, with statistically significant FAME levels comparing the control SCID and the tumor-bearing SCID mice at 4 weeks of age and 17 weeks of age (in Figure S2, all of the data are graphically represented). Palmitic acid (16:0) diminished in the control SCID and the tumor-bearing mice during aging (from 4 weeks of age to 17 weeks of age), along with increases in ARA. MUFA oleic acid (9c-18:1) decreased during aging (from 4 weeks to 17 weeks) in both the control and the tumor-bearing mice. ARA in tumor-bearing mice increased significantly, compared with control SCID, and oleic acid diminished, comparing the 4-week-old and 17-week-old mice of both series.

From the FAME quantities, relevant fatty acid calculations, such as sums, ratios, and equations could be applied, such as SFA, MUFA, PUFA, SFA/MUFA, SFA/PUFA, ω6/ω3, the unsaturation index (UI), and the peroxidation index (PI). Table 3 (see Table S8 for *p*-values) shows these data in triplicate for each mice group in this work: control SCID mice and tumor-bearing SCID mice at 4 weeks of age and 17 weeks of age. The above reported changes in FAME influenced the total SFA and PUFA ω6 levels, accordingly (SFA control; *p* = 0.0051 and tumor-bearing; *p* = 0.0112. PUFA ω6 control; *p* = 0.0053; tumor-bearing; *p* = 0.0152).

The calculations for the unsaturation index (UI) and the peroxidation index (PI) were carried out by Equations (1) and (2), respectively:

UI = (%MUFA × 1) + (%LNA × 2) + (%DGLA × 3) + (%ARA × 4) + (%EPA × 5) + (%DHA × 6)(1)

PI = (%MUFA × 0.025) + (%LNA × 1) + (%DGLA × 2) + (%ARA × 4) + (%EPA × 6) + (%DHA × 8)(2)

**Figure 6:**
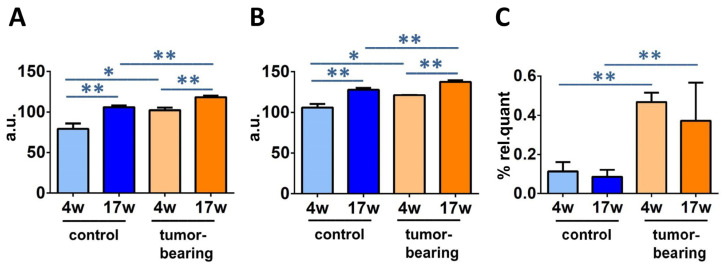
Significantly different fatty acid indices and families in SCID mice cohorts at different ages: (**A**) unsaturation index (UI); (**B**) peroxidation index (PI); (**C**) total TFA (for specific values, see Table 3). Significance: * (*p* < 0.05), ** (*p* < 0.01).

UI and PI indicate the content of unsaturated lipids that impact the membrane properties, as MUFA and PUFA double bonds, and the chemical oxidative reactivity, mainly as PUFA double bonds, respectively [45,46]. The PI and UI values for control and tumor-bearing mice are reported in Table 3 (see Table S8 for the *p*-values) and are graphically reported in Figure 6A, B, respectively.

By following the formation of TFA, it is possible to identify a peculiar transformation of the natural cis unsaturated fatty acids into their geometrical isomers catalyzed by free radicals [33,34,47]. Figure 6C shows a statistical enhancement of TFA, in particular mono-trans ARA (see Figure S2), observed in young and older xenografted animals, compared with control SCID (4 weeks of age; *p* = 0.004, 17 weeks of age; *p* = 0.0124). It is worth noting that the free radical stress, expressed by the formation of TFA, reached the highest level in older mice, regardless of whether or not they were tumor-bearing.

When normal Swiss mice at 4 weeks of age and 17 weeks of age were used to isolate brain lipids and to identify the fatty acid pool differences, we noticed that a few significantly different values were found by comparing the two ages, i.e.,: the increase of palmitic acid (*p* = 0.0011) and the ω6/ω3 ratio (*p* = 0.048), the decrease in the PUFA ω3 EPA (*p* = 0.0040), and the decrease in the total TFA (*p* = 0.0417) (see Table S9). A comparison between the normal Swiss mice and the control SCID mice was also carried out in order to obtain valuable information related to immunodeficiency and radical-based reactivity in the brain tissue which was, especially useful in evaluating the age effect (Table S10). A significant reduction in SFA and an increase in PUFA ω3 and total PUFA was observed in 17-week-old SCID, compared with normal mice (SFA; *p* = 0.0256, ω-3; *p* = 0.0190, PUFA; *p* = 0.0087). Furthermore, the SFA/MUFA and SFA/PUFA ratios were statistically decreased (SFA/MUFA; *p* = 0.0442, SFA/PUFA; *p* = 0.0132) and the indices of unsaturation and peroxidation were enhanced (UI; *p* = 0.0112, PI; *p* = 0.0045). Significantly higher levels of DHA and lower levels of palmitic acid were also found for 17-week-old SCID animals (DHA; *p* = 0.0072, 16:0; *p* = 0.0009). Although further differences of the membrane fatty acid content between the two groups were present, such as total TFA and EPA decreases in younger mice and increases in older ones, as well as MUFA increases at both ages, there were no significances among these distinct conditions. In the younger animals, only 9t-18:1 was significantly reduced in 4-week-old diseased mice, compared to healthy mice of the same age (*p* = 0.0221, Table S10).

## 4. Discussion

Brain is a tissue with one of the highest levels of oxidative metabolism that is consistently associated with the production of oxidative damage. Some studies indicate that specific brain regions affect the responses to DNA damage, with single strand breaks (SSB) considered to be a serious threat to the aging brain [48]. Moreover, there is a strong connection between brain mitochondrial ROS production, glutamate excitotoxicity, and neuronal cell damage [49]. High ROS production, severe DNA damage, inflammatory stress, and heterochromatinization were found in postmitotic neurons from old C57Bl/6 mice, revealing that mature neurons develop a senescence-like phenotype with aging [50]. DNA damage increases with age and in multiple neurological and neurodegenerative disorders, such as Alzheimer’s disease, which is a disorder that is characterized by the accumulation of double strand breaks (DSB) in both neuronal and glial cells [51], as well as a diminution in the expression of UDG1, polb, and bOGG1 glycosylases in AD brains, compared with age-matched controls [52]. Apart from these enzymes and the BER pathway, NER also participates in the progression of age-related cognitive decline, as accumulating DNA damage and reduced synaptic plasticity were observed in the hippocampus of *Ercc1*^Δ/−^ mice [53].

cPu lesions are unique lesions that are generated by H-atom abstraction from the C5′–H position of purine moieties by HO^•^ radicals, eventually resulting in the formation of an additional C5′−C8 covalent bond [4,5,6,54]. On the other hand, 8-oxo-Pu lesions are derived from oxidation at the C8 position of purine moieties by a variety of ROS, such as H_2_O_2_, singlet oxygen, and ONOO^−^, in addition to HO^•^ radical [55,56]. The observation of lesions is connected with the efficiency of the repair. Damaged DNA activates different responses in a cellular environment, depending on the kind of damage, and cells may undergo apoptosis to avoid the propagation of defective cells. However, robust DNA repair and damage-bypass mechanisms protect the stability of the human genome, either by removing the damage or permitting the damage to ensure genome integrity. Together with determining the levels the oxidatively-induced 8-oxo-Pu, we determined the levels of cPu lesions. Both cPu and 8-oxo-cPu formation were evaluated in this study in young and old immunodeficient mice, with and without tumor implantation, to compare the effect of tumor development and to gather information on the aging process. This work is a continuation of an exploratory study of two groups of mice that were examined for the accumulations of liver and kidney DNA lesions [37]. In the present study, we deepened the oxidative DNA damage of the brain, as this tissue maintains a particularly high basal metabolic rate to fulfill the high-energy demand and produces increased levels of ROS [52]. Our results showed that the levels of the four cPu and 8-oxo-dA lesions are similar, in the range of 0.1–0.25 lesions/10^6^ Nu, whereas 8-oxo-dG is one order of magnitude higher, independently of being SCID control, tumor-bearing SCID or normal Swiss mice in their lifetimes (4 weeks old or 17 weeks old) (Figure 3 and Table S1). In the cPu series, the following order was observed in all cases: 5′*S*-cdG > 5′*R*-cdG > 5′*R*-cdA > 5′*S*-cdA.

The detection of cPu in brain tissue was also reported by Wang et al., who analyzed the levels of these adducts in the brain of wild type and *Ercc1^−/Δ^* mice of different ages using capillary high-performance liquid chromatography-tandem mass spectrometry [57]. No significant differences were found between wild type animals of 10 weeks, 21 weeks, and 3 years of age, with the levels of the four cPu lesions being in the range of 0.1–0.5 lesions/10^6^ Nu. On the other hand, a diminution of all four cPu lesions was found in 21-week-old *Ercc1^−/Δ^* mice, compared with 10-week-old mice. Furthermore, it should be noted that the significantly raised amounts of 5′*S*-cdG in 10-week-old deficient-progeroid animals compared to wild-type animals of the same age demonstrated the role of ERRC-1 protein in NER involvement [57]. cdA was also present in the brains of the XP group A gene-knockout (Xpa^−/−^) and significant accretion were detected in mice at 6, 24, and 29 months of age, compared to wild-type animals, through an improved enzyme-linked immunosorbent assay (ELISA) [15] using a novel monoclonal antibody (cdA-1) specific for cdA in single-stranded DNA [58]. However, the cdA levels in the brain tissue of 4-week-old (one month), 12-week-old (three months), and 24-week-old (6 months) mice were fully raised through ELISA quantification, with detectable amounts of ~3.5–4/10^6^ [15]. Immunoassays methods are characterized by adequate simplicity and reproducibility and they can be applied to DNA adduct detection, such as screening the lesions of 8-oxo-2′-deoxyadenosine [59], benzo[a]pyrene-dioleperoxide [60], and 4-hydroxyequilenin [61]. Competitive approaches with the presence of monoclonal antibodies offer higher specificity and effectiveness [58]. On the other hand, only chromatography-based methodologies coupled with isotope dilution mass spectrometry can provide the structural specificity of the studied adduct and the detailed molecular composition for accurate quantification [62,63].

The diastereomeric ratio (5′*R*/5′*S*) can be an important indicator for mechanistic issues that are related to structural conformation of both isomeric forms in association with their abundance and repair. cdA and cdG lesions are excised with similar efficiency by NER machinery in human HeLa cell extracts; however, the 5′*R*-diastereoisomers of both cdA and cdG cause greater distortion of the DNA backbone, thereby being better substrates of NER than the corresponding 5′*S* ones [7]. As shown in Figure 4C, *R* is always more abundant than *S* in the cdA in each group of mice, whereas in the cdG, *S* always exceeds the *R* diastereoisomer. Another important aspect is derived by comparing these diastereomeric ratios between young and older organisms. Lower level of *R*/*S* cdG and cdA ratios were depicted in 17-week-old brain tissues of both control and tumor-bearing animals compared to 4-week-old tissues (Table S5). Significantly raised levels of cdA and cdG in both the *R* and *S* isoforms were also found in the brain tissues of the Long-Evans Cinnamon (LEA) rat, an animal model for human Wilson’s disease, and the Long-Evans Agouti rat (LEC), healthy rats, using NanoLC-NSI-MS/MS analysis [64]. Although the *R*/*S* ratios constitute an index of cPu lesions’ repair efficiency, differences in analytical procedures across distinct research groups did not provide a clear scenario for *R*/*S* formation and biological significance. Previous studies through LC/MS or GC/MS showed a high accumulation of cPu in knockout mice, such as both cdG and cdA in the brains of prdx1^−/−^ mice [65]. Increased levels of 5′*S*-cdA were measured in different organs of wild-type and csb knockout mice, with a significant enrichment of this unrepaired adduct in the brains of csb(-/-) animals, as well as in the livers and the kidneys, suggesting the important role of CSB protein in the DNA repair process [66].

**Figure 7:**
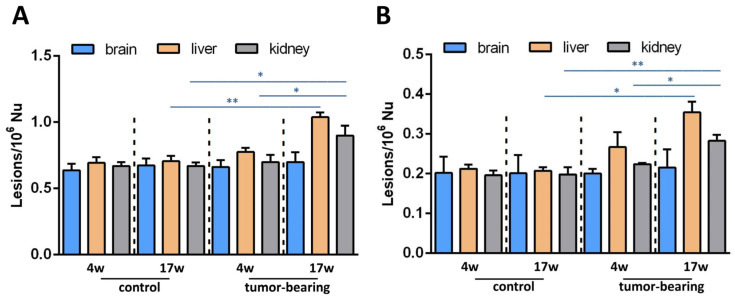
(**A**) cPu levels and (**B**) 8-oxo-dA levels in the brain tissue of control SCID and tumor-bearing SCID mice compared with previous tissue-specific patterns from the liver and the kidney [37] by isotope dilution liquid chromatography-tandem mass spectrometry * (*p* < 0.05), ** (*p* < 0.01).

At this point, it is worth mentioning that we reported an earlier detection of the four cPu levels of liver and kidney tissues at initial and final stages of tumorigenesis in the same animal cohorts (4-week-old and 17-week-old tumor-bearing mice, respectively) [37]. In both tissues of 17-week-old xenografts, we found increased cPu levels. In Figure 7A, a comparison of the total cPu lesions in brain, liver, and kidney tissues is presented. Unlike in brain tissue, statistically significant alterations were exhibited for total cPu in liver and kidney tissues during cancer progression, as highlighted (see also Table S11). A significant enhancement of cPu was found in the liver tissue of 17-week-old control mice, compared with 17-week-old tumor-bearing SCID mice (*p* = 0.0014, Table S12). Furthermore, a comparison between 4-week-old and 17-week-old xenografted animals showed a significant increase of cPu in kidney tissues (*p* = 0.00395). Higher levels of cPu were also found in the kidney tissues of 17-week-old control mice, compared with tumor-bearing SCID mice of the same age (*p* = 0.0365,). 8-oxo-dA levels were previously reported in liver and kidney tissues, and here we measured this lesion in brain tissue. Figure 7 shows the much lower extent of this lesion in the brain, compared with the other tissues. The data presented in our previous [37] and present exploratory studies indicate a differential involvement of genomic instability in mice cohorts, and may contribute to the ongoing debate about the central role of DNA and genome instability in the aging process, which also implies choosing the best biomarkers for such monitoring [67].

A parallel examination of brain lipids can widen the perspectives in the assessment of aging and health conditions, such as tumoral progression, offering opportunities to obtain the holistic approach that is needed to fully understand complex organisms. Our results can be coupled with our previously reported data on the same mice cohorts, which were examined for their erythrocyte membrane phospholipid contents [37]. Evaluating mice brain fatty acid-containing lipids, our data are concerned mostly with glycerophospholipids, which reach almost 70% of the total lipid composition in this tissue [68]. This is the lipid class where the fatty acid composition is influenced by the so-called remodeling mechanism and reports the systemic effects of fatty acids introduced by different diets or changed in response to life conditions [34,36,69,70]. We are aware that the powerful shotgun lipidomic analysis is used to go into the details of all lipid classes [71]; however, it is worth emphasizing that by using the gas chromatographic analysis, we can obtain a precise and reliable separation of geometrical and positional fatty acid isomers, and individuate trans fatty acids that are crucial indicators of free radical stress (thiyl radicals, in particular) [32,33,72]. The role of oxygen in the formation of cPu was recently investigated in cellular models of defective CSA- and CSB-transformed fibroblasts and their normal counterparts, cultured under various oxygen tensions and increased levels of cPu, were detected under hypoxic conditions in both CSA- and CSB-defective cell lines, compared with normal cells [13]. In the same cellular models, the analysis of fatty residues in membrane phospholipids was studied, due to oxygen tensions [42]. A parallel examination of purine adduct formation and membrane molecular transformations in human embryonic epithelial cells, silenced for XPA under hyperoxic conditions, was performed, showing that oxygen promotes enzymatic transformations of the fatty acid pool [12].

Two results can be highlighted in the brain remodeling process: (a) the decrease in palmitic acid accompanied with the increase in arachidonic acid in both groups of immunodeficient (SCID) mice and tumor-bearing SCID mice, comparing 4-week-old to 17-week-old mice (cf. Figure 5); and (b) the increases in UI, PI, and total TFA going from 4-week-old control SCID mice to the tumoral groups (cf. Figure 6). The first data point attention to brain-signaling that starts from membrane phospholipids, and to the detachment of fatty acids for the production of bioactive lipids.The ARA membrane enrichment can lead to the disruption of an equilibrium with the omega-3 counterparts, especially DHA, which regulates neuroinflammation [20]. In control SCID mice, DHA is actually increased, whereas it diminishes in tumor-bearing animals, involving complications when disease conditions add to the aging process. It is worth highlighting that the ARA increase was also reported in the erythrocyte membrane analysis of the same animal cohorts [37], confirming the reporting role of this blood cell for a systemic condition. The combination of lipid metabolism and inflammation is also important in the development of therapeutical strategies for aging and diseases [73]. The second set of data, about UI and PI increases, is connected with an oxidatively-prone lipid environment that is, indeed, in connection with aging and disease progression. As discussed in the Introduction, neurodegeneration is strictly connected as a cause or as a consequence of oxidative reactivity [19,21,29], which is favored by the enrichment of PUFA, as seen in our animal cohorts. It is worth stating that no dietary changes were applied to the animals and that the old tumor-bearing animals were characterized by poor health conditions, so that the PUFA increase in the brain surely did not derive from higher intakes. These observations, together with information about increased TFA in tumor-bearing mice, are relevant in determining that in this particular health condition, the formation of radical reactive species, such as thiyl radicals, occurs, because the formation of TFA is a marker of this specific reactivity [47]. We can recall that thiol-disulfide homeostasis is a very important element of brain metabolism, starting from the hydrogen sulfide production in this tissue [74]. The overall scenario obtained by this study of DNA and membrane data indicates that in the brain, unlike in the liver and the kidney tissues, molecular changes of aging and disease progressions caused by free radicals occur mostly at the level of lipidome, rather than involving all molecular inventory (DNA in particular). Our results highlight that more research is needed to gain combined information on free radical reactivity in different cellular compartments, using both in vitro and in vivo models. Moreover, due to the analytical conditions that were used in this study, it was not possible to define whether specific areas of the brain were most involved in the effects of the aging or disease, as is well known in differential evaluations [75].

## 5. Conclusions

In the present work, the simultaneous measurement of two important molecular contributions, genetic (DNA) and lipid components, was performed under specific conditions of aging and disease in a murine model, completing the picture with information from brain tissue. In the brain tissue of young and older tumor-bearing animals, the analysis of four cPu and two 8-oxo-Pu lesions showed a slight but significant progressive age-dependent accumulation only for the 5′*S*-cdA and 5′*S*-cdG lesions. cPu were detected in various tissue types and clinical specimens and did not suffer from stability issues and artifacts, unlike the 8-oxo-Pu that are formed by oxidizing species. At the same time, fatty acid pool remodeling due to aging and tumoral conditions occurred, specifically involving SFA and PUFA, with TFA as biomarker of free radical stress. The overview of the molecular contributions of DNA and lipids adds new insights into the consequences of aging and disease, highlighting the brain’s prevalent lipid remodeling response and inflammatory signaling, which seem to prevail over the effects of DNA damage. These results can inspire further and deeper in vitro and in vivo model investigations on protective and therapeutic strategies of neurodegenerative disorders, taking into account the extensive involvement of the membrane lipids seen in our model. Under aging and disease progressions, membranes are not spectators [76,77]. Membrane-targeted strategies tailored to the specific lipidome profile are needed to preserve the molecular integrity of the membrane and to demonstrate the effects of delaying degenerative processes as a whole.

## Figures and Tables

**Table 1:** The levels (lesions/10^6^ nucleosides) of 5′*R*-cdG, 5′*S*-cdG, 5′*R*-cdA, 5′*S*-cdA, 8-oxo-dG, and 8-oxo-dA in the brain tissues of control SCID and tumor-bearing SCID, 4 weeks of age- and 17 weeks of age, respectively (mean ± standard deviation of six sample measurements).

	5′*R*-cdG	5′*R*-cdA	5′*S*-cdG	5′*S*-cdA	8-oxo-dG	8-oxo-dA
control 4w	0.176 ± 0.011	0.140 ± 0.016	0.205 ± 0.028	0.116 ± 0.014	1.828 ± 0.183	0.202 ± 0.041
control 17w	0.179 ± 0.036	0.146 ± 0.019	0.211 ± 0.024 ^1,^*	0.137 ± 0.012 ^2,^*	1.783 ± 0.208	0.201 ± 0.046
tumor-bearing 4w	0.185 ± 0.018	0.153 ± 0.014	0.213 ± 0.034	0.112 ± 0.014	1.730 ± 0.276	0.201 ± 0.012
tumor-bearing 17w	0.178 ± 0.031	0.140 ± 0.020	0.245 ± 0.025 ^3,^*	0.136 ± 0.012 ^3,^*	1.903 ± 0.234	0.216 ± 0.046

^1^ Comparison between control 17-week-old mice vs. tumor-bearing 17-week-old mice, ^2^ Comparison between control 4-week-old mice vs. control 17-week-old mice, ^3^ Comparison between tumor-bearing 4-week-old mice vs. tumor-bearing 17-week-old mice. Statistical significance: * (*p* < 0.05).

**Table 2:** Relative quantitative percentages (% rel. quant.) of fatty acid methyl esters (FAME) obtained from brain tissues of control SCID mice and tumor-bearing SCID mice at different ages ^1^.

FAME	FA Family	Control (4w)	Control (17w)	Tumor- Bearing (4w)	Tumor- Bearing (17w)
Palmitic acid (16:0)	SFA	23.67 ± 1.27 *	20.53 ± 0.54 **	25.87 ± 0.60	22.67 ± 0.51 **
Stearic acid (18:0)		26.20 ± 1.77	26.24 ± 0.40	23.58 ± 0.04	24.22 ± 0.88 *
Palmitoleic acid (9c-16:1)	MUFA	0.70 ± 0.25	0.79 ± 0.22	0.86 ± 0.01	0.67 ± 0.14
Oleic acid (9c-18:1)		24.02 ± 0.41	22.96 ± 0.75	20.19 ± 1.60 *	19.32 ± 0.91 **
Vaccenic acid (11c-18:1)		8.01 ± 1.31	6.76 ± 0.83	6.12 ± 0.83	6.09 ± 0.09
LNA (18:2-ω6)		1.02 ± 0.16	0.95 ± 0.12	0.92 ± 0.02	1.14 ± 0.15
DGLA (20:3-ω6)	PUFA ω6	1.18 ± 0.07	0.87 ± 0.21	0.82 ± 0.07	1.04 ± 0.12
ARA (20:4-ω6)		11.06 ± 1.26 **	15.63 ± 0.71 *	14.96 ± 0.07 *	19.62 ± 1.18 **
EPA (20:5-ω3)	PUFA ω3	0.57 ± 0.50	0.53 ± 0.18	1.13 ± 0.29	1.08 ± 0.61
DHA (22:6-ω3)		3.40 ± 0.16 **	4.60 ± 0.20	4.04 ± 0.18 *	3.67 ± 0.65
9t-18:1	TFA	0.03 ± 0.02	0.06 ± 0.04	0.06 ± 0.03	0.10 ± 0.05
mt-ARA 20:4		0.11 ± 0.05	0.09 ± 0.03	0.47 ± 0.05 **	0.37 ± 0.19 *

^1^ The values are given as mean ± SD (*n* = 3). Statistical significances: * (*p* < 0.05), ** (*p* < 0.01).

**Table 3:** Relative quantitative percentages (% rel. quant.) of fatty acid methyl families and ratios/indices obtained from brain tissues of control SCID mice and tumor-bearing SCID mice at different ages ^1^.

FA Family	Index	Control (4w)	Control (17w)	Tumor- Bearing (4w)	Tumor- Bearing (17w)
SFA		49.87 ± 0.50 **	46.77 ± 0.83 *	49.45 ± 0.56	46.89 ± 0.47
MUFA		32.74 ± 1.68	30.51 ± 0.48	27.16 ± 2.44	26.09 ± 0.83 **
PUFA ω6		13.26 ± 1.16 **	17.45 ± 0.61 *	16.70 ± 0.01 *	21.79 ± 1.36 **
PUFA ω3		3.98 ± 0.66	5.13 ± 0.33	5.17 ± 0.46	4.76 ± 0.42
PUFA ^1^		17.24 ± 1.36 **	22.58 ± 0.51 **	21.87 ± 0.45 *	26.55 ± 0.94 **
TFA		0.15 ± 0.04	0.14 ± 0.04	0.52 ± 0.01 **	0.47 ± 0.23 **
	SFA/MUFA	1.53 ± 0.09	1.53 ± 0.05	1.83 ± 0.19	1.80 ± 0.05 **
	SFA/PUFA	2.90 ± 0.22 **	2.07 ± 0.08 **	2.26 ± 0.02 *	1.77 ± 0.08 **
	ω6/ω3	3.39 ± 0.58	3.41 ± 0.29	3.25 ± 0.29	4.62 ± 0.72
	Unsaturation Index (UI)	105.87 ± 4.48 **	127.78 ± 2.38 **	121.17 ± 0.11 *	137.40 ± 2.14 **
	Peroxidation Index (PI)	79.13 ± 6.76 **	105.94 ± 2.22 **	102.15 ± 3.22 *	118.22 ± 1.88 **

^1^ PUFA = % PUFA ω-3+ % PUFA ω-6. Statistical significance: * (*p* < 0.05), ** (*p* < 0.01).

## Data Availability

The data presented in this study are provided in this article and in the Supplementary Materials.

## Supplementary Materials

The following supporting information can be downloaded at: https://www.mdpi.com/article/10.3390/biom12081075/s1, Figure S1: Graphical presentation of of cPu and 8-oxo-Pu lesions in the brain tissues of 4-week-old and 17-week-old normal mice; Figure S2: Graphical presentation of FAME in control SCID mice and tumor-bearing SCID mice at 4 weeks and 17 weeks of age; Tables S1 and S2: The levels of 5′*R*-cdG, 5′*S*-cdG, 5′*R*-cdA, 5′*S*-cdA, 8-oxo-dG, and 8-oxo-dA lesions and statistical analysis in the brain tissues of control SCID, tumor-bearing SCID, and normal Swiss mice; Tables S3 and S4: The levels of cPu and 8-oxo-Pu lesions and statistical analysis in the brain tissues of control SCID, tumor-bearing SCID, and normal Swiss mice; Tables S5 and S6: 5′*R*/5′*S* ratio in DNA-isolated and statistical analysis from the brain tissues of control SCID, tumor-bearing SCID, and normal Swiss mice; Tables S7 and S8: Statistical analysis of fatty acids, families, and lipid indices in the brain tissues of control SCID and tumor-bearing SCID mice at 4 weeks and 17 weeks of age; Tables S9 and S10: Fatty acid methyl esters, families, and lipid indices and statistical analysis in the brain tissues of normal mice at 4 weeks and 17 weeks of age; Tables S11 and S12: cPu and 8-oxo-dA levels and statistical analysis in the brain, liver, and kidney tissue of control SCID, tumor-bearing SCID, and normal Swiss mice at 4 weeks and 17 weeks of age.

## Abbreviations

ARA: arachidonic acid; BER, base excision repair; cdA, 5′,8-cyclo-2′-deoxyadenosine; cdG, 5′,8-cyclo-2′-deoxyguanosine; cPu, purine 5′,8-cyclo-2′-deoxynucleoside; DHA, docosahexaenoic acid; DGLA, dihomo-gamma-linolenic acid; DPA, docosapentaenoic acid; ELO, elongase; EPA, eicosapentaenoic acid; FA, fatty acid; FAME, fatty acid methyl esters; GC, gas chromatography; HPLC, high-pressure liquid chromatography; LC-MS/MS, liquid chromatography tandem mass spectrometry; LNA, linoleic acid; MUFA, monounsaturated fatty acids; NER, nucleotide excision repair; 8-oxo-dA, 8-oxo-7,8-dihydro-2′-deoxyadenosine; 8-oxo-dG, 8-oxo-7,8-dihydro-2′-deoxyguanosine; 8-oxo-Pu, purine 8-oxo-7,8-dihydro-2′-deoxynucleoside; PI, peroxidation index; PUFA, polyunsaturated fatty acids; ROS, reactive oxygen species; SCID, severe combined immunodeficient; SFA, saturated fatty acids; TFA, trans fatty acids; UI, unsaturation index.
