# Supplementary material for: Effects of Aging and Disease Conditions in Brain of Tumor-Bearing Mice: Evaluation of Purine DNA Damages and Fatty Acid Pool Changes
Source: Biomolecules. 2022 Aug 4;12(8):1075. doi: 10.3390/biom12081075 (PMC9405824; doi:10.3390/biom12081075)
Supplement: Supplementary file 1 [file biomolecules-12-01075-s001.zip › biomolecules-1785766-supplementary.pdf]

biomolecules

Supporting Material

Effects of Aging and Disease Conditions in Brain of Tumor-Bearing Mice: Evaluation of Purine DNA Damages and Fatty Acid Pool Changes

Marios G. Krokidis *, Paraskevi Prasinou, Eleni K. Efthimiadou, Andrea Boari,
Carla Ferreri and Chryssostomos Chatgilialoglu *

* Correspondence: m.krokidis@inn.demokritos.gr (M.G.K.); chrys@isof.cnr.it (C.C.)

List of Contents:

Table S1 and Table S2	page S2
Figure S1, Table S3 and Table S4	page S3
Table S5 and Table S6	page S4
Table S7 and Table S8	page S5
Figure S2	page S6
Table S9	page S7
Table S10	page S8
Table S11	page S9
Table S12	page S10

Table S1. The levels (lesions/10^6 nucleosides) of 5'*R*-cdG, 5'*S*-cdG, 5'*R*-cdA, 5'*S*-cdA, 8-oxo-dG and 8-oxo-dA in the brain tissues of control SCID, tumor-bearing SCID and normal Swiss mice, 4-weeks and 17-weeks old respectively (mean ± standard deviation of six sample measurements). Part of this Table is included in Table 1.

	**5'*R*-cdG**	**5'*R*-cdA**	**5'*S*-cdG**	**5'*S*-cdA**
control 4w	0.176±0.011	0.140±0.016	0.205±0.028	0.116±0.014
control 17w	0.179±0.036	0.146±0.019	0.211±0.024	0.137±0.012
tumor-bearing 4w	0.185±0.018	0.153±0.014	0.213±0.034	0.112±0.014
tumor-bearing 17w	0.178±0.031	0.140±0.020	0.245±0.025	0.136±0.012
normal 4w	0.159±0.020	0.140±0.023	0.213±0.025	0.115±0.010
normal 17w	0.164±0.030	0.128±0.020	0.209±0.022	0.117±0.009

	**8-oxo-dG**	**8-oxo-dA**
control 4w	1.828±0.183	0.202±0.041
control 17w	1.783±0.208	0.201±0.046
tumor-bearing 4w	1.730±0.276	0.201±0.012
tumor-bearing 17w	1.903±0.234	0.216±0.046
normal 4w	1.719±0.159	0.184±0.038
normal 17w	1.638±0.281	0.184±0.020

Table S2. The *p* values for cPu and 8-oxo-Pu lesions obtained from the data of Table S1; control is the SCID mice without tumor and normal is the healthy Swiss mice.

	**5'*R*-cdG**	**5'*R*-cdA**	**5'*S*-cdG**	**5'*S*-cdA**	**8-oxo-dG**	**8-oxo-dA**
control 4w vs. control 17w	0.8513	0.5458	0.4729	0.0102*	0.6988	0.9740
tumor-bearing 4w vs. tumor-bearing 17w	0.6827	0.4100	0.0354*	0.0342*	0.1493	0.7445
control 4w vs. tumor-bearing 4w	0.6241	0.4513	0.7234	0.6414	0.2097	0.6828
control 17w vs. tumor-bearing 17w	0.9723	0.3220	0.0370*	0.8887	0.4557	0.6194
normal 4w vs. normal17w	0.5853	0.4522	0.5677	0.6718	0.4447	0.9712
normal 4w vs control 4w	0.0885	0.9578	0.3060	0.8742	0.1858	0.4389
normal 17w vs control 17w	0.5364	0.0716	0.8662	0.0049**	0.3871	0.2169

Statistically significant samples: * <0.05 , **<0.01

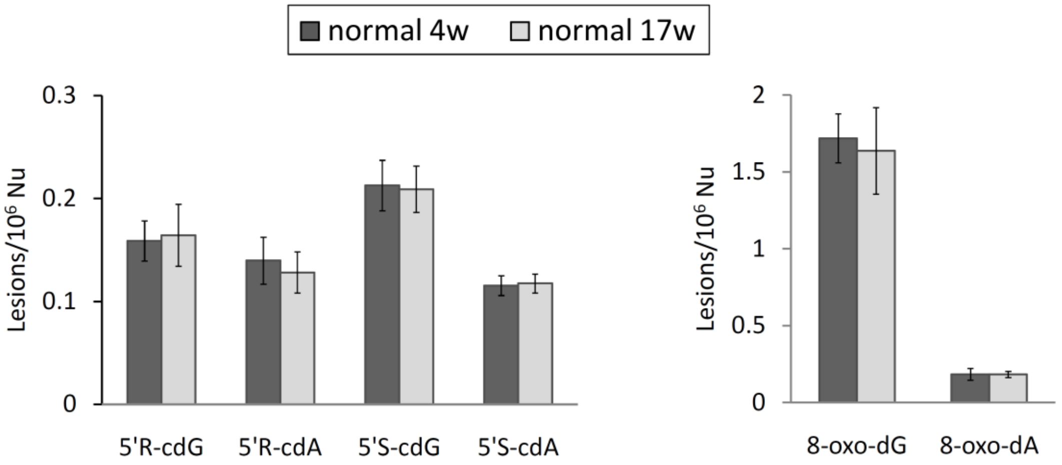

Figure S1. The levels (lesions/10^6 nucleosides) of cPu and 8-oxo-Pu in the brain tissues of 4-weeks and 17-weeks old normal mice. For specific values see Table S1.

Table S3. Total amount of cPu and 8-oxo-Pu (lesions/10^6 nucleosides) in the brain tissues of control SCID, tumor-bearing SCID and normal Swiss mice.

	**cPu**	**8-oxo-Pu**
control 4w	0.637±0.049	2.030±0.188
control 17w	0.674±0.053	1.984±0.206
tumor-bearing 4w	0.662±0.053	1.931±0.241
tumor-bearing 17w	0.699±0.074	2.118±0.250
normal 4w	0.626±0.068	1.903±0.192
normal 17w	0.619±0.064	1.821±0.295

Table S4. The *p* values for the sum of cPu and 8-oxo-Pu lesions obtained from the data of Table S3.

	**cPu**	**8-oxo-Pu**
control 4w vs. control 17w	0.0633	0.6181
tumor-bearing 4w vs. tumor-bearing 17w	0.4808	0.1885
control 4w vs. tumor-bearing 4w	0.1422	0.2579
control 17w vs. tumor-bearing 17w	0.4914	0.4173
normal 4w vs. normal17w	0.7844	0.4419
normal 4w vs control 4w	0.8457	0.1034
normal 17w vs control 17w	0.1001	0.3095

Table S5. 5'*R*/5'*S* ratio in DNA isolated from the brain tissues of control SCID, tumor-bearing SCID and normal Swiss mice.

	**R/S cdG**	**R/S cdA**
control 4w	0.867±0.094	1.224±0.211
control 17w	0.855±0.196	1.076±0.184
tumor-bearing 4w	0.879±0.117	1.375±0.242
tumor-bearing 17w	0.731±0.106	1.031±0.158
normal 4w	0.749±0.069	1.208±0.163
normal 17w	0.788±0.127	1.088±0.122

Table S6. The *p* values for the 5'*R*/5'*S* ratio obtained from the data of Table S5.

	**R/S cdG**	**R/S cdA**
control 4w vs. control 17w	0.8926	0.2059
tumor-bearing 4w vs. tumor-bearing 17w	0.1108	0.0211*
control 4w vs. tumor-bearing 4w	0.8457	0.2242
control 17w vs. tumor-bearing 17w	0.0852	0.0688
normal 4w vs. normal17w	0.4216	0.3024
normal 4w vs control 4w	0.0517	0.9068
normal 17w vs control 17w	0.6203	0.8837

Statistically significant sample: * <0.05

Table S7. The *p* values of fatty acids obtained from the comparison between brain tissues of control SCID and tumor-bearing SCID mice at 4 weeks and 17 weeks after conducting unpaired t-test (see Table 2).

FAME	control 4w vs. control 17w	tumor-bearing 4w vs. tumor-bearing 17w	control 4w vs. tumor-bearing 4w	control 17w vs. tumor-bearing 17w
16:0	0.0169*	0.0074**	0.1158	0.0073**
18:0	0.9694	0.4005	0.1411	0.0225*
9c-16:1	0.6885	0.1797	0.4756	0.4994
9c-18:1	0.0990	0.4828	0.0235*	0.0060**
11c-18:1	0.2337	0.9534	0.1736	0.2379
LNA (18:2-ω6)	0.5710	0.1477	0.4722	0.1642
DGLA (20:3-ω6)	0.0713	0.1205	0.0111*	0.3045
ARA (20:4-ω6)	0.0054**	0.0132*	0.0255*	0.0074**
EPA (20:5-ω3)	0.8910	0.9312	0.2620	0.2084
DHA (22:6-ω3)	0.0013**	0.5126	0.0247*	0.0775
9t-18:1	0.0559	0.3476	0.3419	0.2811
mt-ARA	0.4831	0.5594	0.0040**	0.0124*

Statistically significant samples: * <0.05, **<0.01

Table S8. The *p* values of fatty acid families and lipid indexes obtained from the comparison between brain tissues of control SCID and tumor-bearing SCID mice at 4 weeks and 17 weeks after conducting unpaired t-test (see Table 3).

FA family / Indexes	control 4w vs. control 17w	tumor-bearing 4w vs. tumor-bearing 17w	control 4w vs. tumor-bearing 4w	control 17w vs. tumor-bearing 17w
SFA	0.0051**	0.0112*	0.4351	0.8360
MUFA	0.0915	0.5059	0.0530	0.0013**
PUFA ω-6	0.0053**	0.0152*	0.0285*	0.0073**
PUFA ω-3	0.0535	0.3787	0.1189	0.2918
PUFA	0.0031**	0.0081**	0.0211*	0.0030**
TFA	0.9467	0.7872	0.0013**	0.0038**
SFA/MUFA	0.9088	0.7917	0.0821	0.0029**
SFA/PUFA	0.0037**	0.0036**	0.0308*	0.0091**
ω-6/ω-3	0.9505	0.0891	0.7773	0.0532
Unsaturated Index (UI)	0.0017**	0.0020**	0.0195*	0.0065**
Peroxidation Index (PI)	0.0028**	0.0053**	0.0227*	0.0019**

Statistically significant samples: * <0.05 , **<0.01

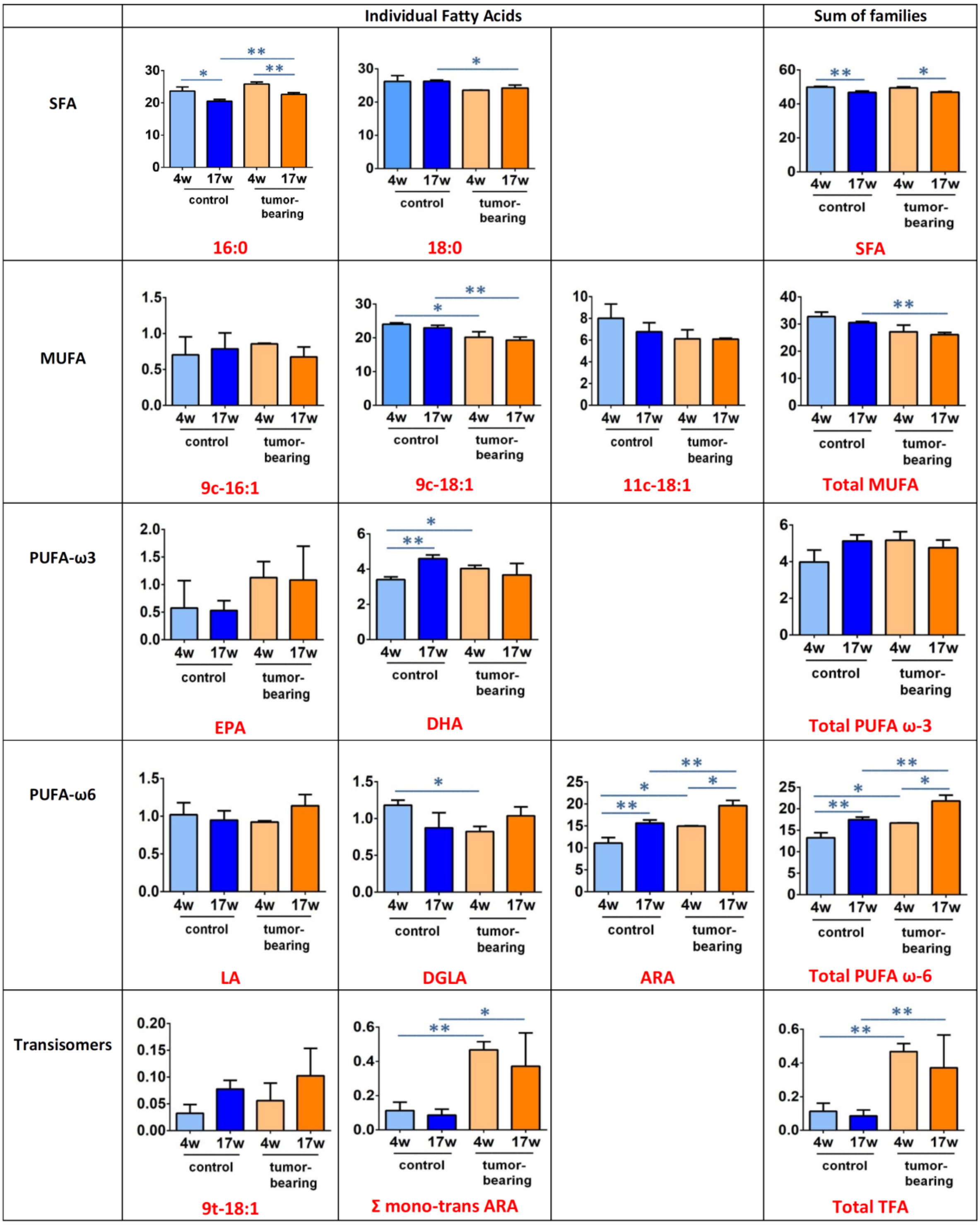

Figure S2. Percentage differences for each type of fatty acid in the brain tissue among control SCID mice and tumor-bearing SCID mice at 4 weeks and 17 weeks of age. The values are given as mean ± SD (n=3). Each member of the fatty acid family is given in a row, the last column being the sum of the corresponding fatty acid family. * ($p < 0.05$), ** ($p < 0.01$); For specific values see Tables 2, 3, S7 and S8.

Table S9. Relative percentages (% rel) of fatty acid methyl esters (FAME), total FAME per family and lipid indexes obtained from brain tissue of normal mice at different age points (4 weeks and 17 weeks). [1] *p* value represents the comparison between 4-weeks and 17-weeks normal mice after conducting unpaired t-test.[2]

FAME	FA family / Indexes	normal (4w)	normal (17w)	*p* value
16:0		26.34 ±0.19	28.50 ±0.40	0.0011**
18:0		24.81 ±0.22	24.40 ±1.52	0.6712
9c-16:1		0.71 ±0.09	0.80 ±0.15	0.3977
9c-18:1		21.48 ±1.80	19.96 ±0.54	0.2337
11c-18:1		7.24 ±0.52	7.13 ±0.43	0.8065
LNA (18:2-ω6)		0.95 ±0.07	0.80 ±0.17	0.2347
DGLA (20:3-ω6)		1.03 ±0.13	0.83 ±0.10	0.0985
ARA (20:4-ω6)		13.04 ±1.30	13.89 ±0.41	0.3395
EPA (20:5-ω3)		0.69 ±0.27	0.23 ±0.10	0.0040**
DHA (22:6-ω3)		3.45 ±0.26	3.35 ±0.14	0.5758
9t-18:1		0.06 ±0.02	0.04 ±0.01	0.1138
mt-ARA		0.20 ±0.08	0.07 ±0.01	0.0533
	SFA	51.15 ±0.15	52.90 ±1.18	0.0631
	MUFA	29.42 ±1.98	27.89 ±1.09	0.3061
	PUFA ω-6	15.02 ±1.37	15.52 ±0.33	0.5687
	PUFA ω-3	4.14 ±0.46	3.58 ±0.24	0.0551
	PUFA	19.16 ±1.76	19.10 ±0.15	0.9563
	TFA	0.27 ±0.09	0.11 ±0.03	0.0417*
	SFA/MUFA	1.74 ±0.12	1.90 ±0.12	0.1767
	SFA/PUFA	2.68 ±0.25	2.77 ±0.08	0.6070
	ω-6/ω-3	3.64 ±0.24	4.35 ±0.37	0.0480*
	Unsaturated Index (UI)	110.72 ±5.61	108.79 ±1.50	0.5945
	Peroxidation Index (PI)	87.65 ±8.05	86.89 ±0.57	0.8784

[1] The values are obtained from the GC analysis of FAME, derived from membrane phospholipids as reported in the experimental part. The numbers in the boxes represent the mean value (± standard deviation) of fatty acids from the measurement of three samples per group. [2] Statistically significant samples: * <0.05, **<0.01

Table S10. The *p* values of fatty acids, families and indexes obtained from the comparison between brain tissues of normal Swiss mice and control SCID mice (*p* values obtained from the data of Tables 2, 3 and S9).

FAME	normal Swiss 4w vs. control SCID 4w	normal Swiss 17w vs. control SCID 17w
16:0	↓, *p*=0.0633	↓, *p*=0.0009***
18:0	↑, *p*=0.3241	↑, *p*=0.2356
9c-16:1	↑, *p*=0.9783	↑, *p*=0.9295
9c-18:1	↑, *p*=0.1820	↑, *p*=0.0567
11c-18:1	↑, *p*=0.5360	↓, *p*=0.3548
LA (18:2-ω6)	↑, *p*=0.6410	↑, *p*=0.4397
DGLA (20:3-ω6)	↑, *p*=0.3142	↑, *p*=0.8106
ARA (20:4-ω6)	↓, *p*=0.1820	↑, *p*=0.0797
EPA (20:5-ω3)	↓, *p*=0.8173	↑, *p*=0.1465
DHA (22:6-ω3)	↓, *p*=0.8476	↑, *p*=0.0072**
9t-18:1	↓, *p*=0.0221*	↑, *p*=0.5782
mt-ARA	↓, *p*=0.0721	↑, *p*=0.3065
SFA	↓, *p*=0.0754	↓, *p*=0.0256*
MUFA	↑, *p*=0.2273	↑, *p*=0.0633
PUFA ω-6	↓, *p*=0.2025	↑, *p*=0.0538
PUFA ω-3	↓, *p*=0.8207	↑, *p*=0.0190*
PUFA	↓, *p*=0.3403	↑, *p*=0.0087**
TFA	↓, *p*=0.0562	↑, *p*=0.2724
SFA/MUFA	↓, *p*=0.1912	↓, *p*=0.0442*
SFA/PUFA	↑, *p*=0.4362	↓, *p*=0.0132*
ω-6/ω-3	↓, *p*=0.5059	↓, *p*=0.0775
Unsaturation Index (UI)	↓, *p*=0.4522	↑, *p*=0.0112*
Peroxidation Index (PI)	↓, *p*=0.3975	↑, *p*=0.0045**

Statistically significant samples: * <0.05, **<0.01, ***<0.001

Table S11. cPu and 8-oxo-dA levels in the brain tissue of 4-weeks and 17-weeks of age of control SCID, tumor-bearing SCID and normal Swiss mice compared with previous tissue-specific patterns from the liver and kidney by isotope dilution liquid chromatography-tandem mass spectrometry.

Tissue	control SCID	tumor-bearing SCID	Normal Swiss	Reference
**cPu**				
brain 4w	0.64±0.05	0.66±0.05	0.63±0.07	this study
brain 17w	0.67±0.05	0.70±0.07	0.62±0.06	this study
liver 4w	0.69±0.04	0.78±0.03	0.54±0.08	[37]
liver 17w	0.71±0.04	1.04±0.04	0.55±0.03	[37]
kidney 4w	0.67±0.03	0.70±0.05	0.49±0.00	[37]
kidney 17w	0.67±0.03	0.90±0.07	0.52±0.02	[37]
**8-oxo-dA**				
brain 4w	0.20±0.04	0.20±0.01	0.18±0.04	this study
brain 17w	0.20±0.05	0.22±0.05	0.18±0.02	this study
liver 4w	0.21±0.01	0.27±0.04	0.16±0.02	[37]
liver 17w	0.21±0.01	0.35±0.03	0.18±0.00	[37]
kidney 4w	0.20±0.01	0.22±0.00	0.13±0.01	[37]
kidney 17w	0.20±0.02	0.28±0.01	0.16±0.02	[37]

Table S12. The *p* values for cPu and 8-oxo-dA lesions obtained from Table S11.

	**Brain**	**Liver** [37]	**Kidney** [37]
**cPu**			
control 4w vs. control 17w	0.0633	0.8071	0.9626
tumor-bearing 4w vs. tumor-bearing 17w	0.4808	0.0704	0.0395*
control 4w vs. tumor-bearing 4w	0.1422	0.1484	0.3263
control 17w vs. tumor-bearing 17w	0.4914	0.0014**	0.0365*
**8-oxo-dA**			
control 4w vs. control 17w	0.9740	0.6768	0.9123
tumor-bearing 4w vs. tumor-bearing 17w	0.7445	0.0570	0.022*
control 4w vs. tumor-bearing 4w	0.6828	0.293	0.065
control 17w vs. tumor-bearing 17w	0.6194	0.012*	0.003**

Statistically significant samples: * <0.05 , **<0.01
